# Predicting Chronic Kidney Disease Using Hybrid Machine Learning Based on Apache Spark

**DOI:** 10.1155/2022/9898831

**Published:** 2022-02-23

**Authors:** Manal A Abdel-Fattah, Nermin Abdelhakim Othman, Nagwa Goher

**Affiliations:** ^1^Department of Information Systems, Faculty of Computers and Artificial Intelligence, Helwan University, Cairo, Egypt; ^2^Faculty of Informatics and Computer Science, British University, Egypt, Cairo, Egypt; ^3^Department of Information Systems, Faculty of Computer Science, Nahda University in Beni Suef, Beni Suef, Egypt

## Abstract

Chronic kidney disease (CKD) has become a widespread disease among people. It is related to various serious risks like cardiovascular disease, heightened risk, and end-stage renal disease, which can be feasibly avoidable by early detection and treatment of people in danger of this disease. The machine learning algorithm is a source of significant assistance for medical scientists to diagnose the disease accurately in its outset stage. Recently, Big Data platforms are integrated with machine learning algorithms to add value to healthcare. Therefore, this paper proposes hybrid machine learning techniques that include feature selection methods and machine learning classification algorithms based on big data platforms (Apache Spark) that were used to detect chronic kidney disease (CKD). The feature selection techniques, namely, Relief-F and chi-squared feature selection method, were applied to select the important features. Six machine learning classification algorithms were used in this research: decision tree (DT), logistic regression (LR), Naive Bayes (NB), Random Forest (RF), support vector machine (SVM), and Gradient-Boosted Trees (GBT Classifier) as ensemble learning algorithms. Four methods of evaluation, namely, accuracy, precision, recall, and F1-measure, were applied to validate the results. For each algorithm, the results of cross-validation and the testing results have been computed based on full features, the features selected by Relief-F, and the features selected by chi-squared feature selection method. The results showed that SVM, DT, and GBT Classifiers with the selected features had achieved the best performance at 100% accuracy. Overall, Relief-F's selected features are better than full features and the features selected by chi-square.

## 1. Introduction

The present era, especially the last two decades, can be named the era of big data where digital data is turning out to be very crucial more and more in various fields such as science, healthcare, technology, and society. Huge data volumes have been produced and generated from multiple sensor networks and mobile applications in almost all fields, including healthcare in specific, and this multitude of data volumes is what we call big data [1]. Wide variety of data sources such as streaming machines, high-end output instruments, visualizing, and knowledge extraction across these vast and diverse types of data pose a significant challenge when sufficient cutting-edge technologies and tools are not used. One of the most eminent technological challenges facing big data analytics lays in exploring ways that are adequate to obtain useful and relevant information for different user categories in an effective manner.

Nowadays, the different forms and types of data sources in healthcare are being gathered in both clinical and nonclinical environments, where the most crucial data in healthcare analytics is the digital copy of a patient's medical history. On that account, the process of designing and making up a distributed data system to handle big data is challenged by three main issues. The first challenge is that it is difficult to collect data from distributed locations because of the diverse and large data volume. The second challenge is that storage is the chief issue for heterogeneous and enormous datasets as big data system requires to store while allowing performance guarantee. The third challenge is more connected to big data analytics, specifically to enormous mining datasets in real time, and this includes visualization, prediction, and optimization [2].

Considering the difficulty imposed by these challenges, they require an up-to-date and advanced processing paradigm provided that the present data management systems do not provide adequate efficiency in handling the heterogeneous nature of data or the real-time aspect. Traditional database management systems cannot support the continuous increase in huge data size. To address these issues related to enormous and heterogeneous data storage, the research community has proposed a number of research works, such as Apache Spark, Apache Hadoop [3], Apache Kafka [4], and Apache Storm [5], to solve healthcare problems [6–8].

Chronic kidney disease (CKD) has received a lot of interest due to its high death rate. Chronic diseases have become a major hazard to emerging countries, according to the World Health Organization (WHO) [9]. CKD is a kidney illness that can be treated in its early stages, but it eventually leads to renal failure if not treated early. In 2016, chronic kidney disease claimed the lives of 753 million individuals globally, accounting for 336 million male deaths and 417 million female deaths [10]. Chronic renal disease can be prevented from progressing to kidney failure if diagnosed and treated early. Diagnosing chronic kidney disease early is the best method to treat it, while delaying treatment until it is too late may lead to renal failure, which necessitates dialysis or kidney transplantation to live normally. Therefore, global strategies for early detection and treatment of people with CKD are required. To mine hidden patterns from data for effective decision-making and to help doctors in making more accurate diagnoses, a computer-aided diagnosis system based on artificial intelligence strategies is needed for clinical information. Artificial intelligence techniques (machine learning and deep learning) have been used in the health field, namely, in disease prediction and diagnosis.

Chronic kidney disease (CKD) is a condition that affects the kidney's ability to function. In general, CKD is separated into phases, with renal failures occurring when the kidneys are no longer able to complete their roles of blood purification and mineral balance in the body [11]. According to the current estimates, CKD is more common in adults over 65 years old (38%) than in people aged 45–64 years (12%) and people aged 18–44 years (6%). Women have a rather higher rate of CKD (14%) than males [12].

Machine learning is an exciting field that focuses on studying huge amounts of data with multiple variables. Machine learning has basically developed from studying the theory of pattern recognition and computational learning in artificial intelligence; it presupposes computational methods, algorithms, and analysis techniques. From the perspective of Medical Sciences, machine learning undertakes to aid health specialists and doctors in carrying out scintillate and flawless diagnoses, choosing the best-fit medicines for patients, determining patients at high risk, and, most importantly, improving patients' physical condition with minimal cost.

Machine learning (ML) has demonstrated remarkable performance across a range of applications, such as speech recognition [13], computer vision [14], medical diagnostics [15], and engineering [16].

Being a constituent of the ML process, feature selection (FS) is a crucial preprocessing step that determines the most relevant attributes within a dataset. Removing unimportant and unnecessary attributes can result in less complicated and more accurate models. In this paper, two feature selection methods based on Apache Spark are used, namely, Relief-F [17] and chi-squared [18] feature selection method. Some of the research works have used ML techniques to predict CKD. For example, Charleonnan [19] et al. used four ML algorithms, K-nearest neighbors (KNN), support vector machine (SVM), logistic regression (LR), and decision tree (DT), to predict CKD. Other research works used hybrid ML algorithms that are integrated between feature selection methods and ML to predict CKD. Feature selection methods have been used to reduce the number of features and select the optimal subsets of features from the dataset. For example [20], authors used chi-square, correlation-based feature selection (CFS), and Lasso feature selection to select the essential features from the database. They applied artificial neural network (ANN), C5.0, LR, SVM, KNN, and RF to both full features and the selected features.

Recently, researchers have been using big data platforms such as Apache Spark [21] which is a large-scale data processing engine with a unified analytics engine. Spark is 100 times quicker than Hadoop in running workloads on large-scale clusters. It includes Java, Scala, *Python*, and *R* high-level APIs, as well as an efficient engine that supports broad execution graphs. It also includes a number of higher-level tools such as Spark SQL for SQL and structured data processing, MLlib, GraphX, and Structured Streaming.

Spark's machine learning (ML) [21] library is called MLlib. Its purpose is to make scalable and simple machine learning a reality. It provides, at a high level, tools such as classification, regression, clustering, and collaborative filtering as examples of machine learning algorithms. It also provides feature extraction, transformation, dimensionality reduction, and selection as examples of featurization.

The previous studies of CKD prediction have not used big data platforms to solve this problem. The goal of this work is to predict CKD using hybrid ML techniques based on Apache Spark to predict CKD. Our contribution can be summarized as follows:

  Developing hybrid ML techniques based on Apache Spark to predict CKD
  Applying feature selection algorithms to select the important features from the dataset
  Applying optimization techniques, including grid search with cross-validation to optimize ML algorithms to enhance performance
  Applying different ML classification algorithms to both full features and the selected features
  Applying ensemble learning such as Gradient-Boosted Trees based on Apache Spark to predict CKD.

The rest of this paper is structured as follows: Section 2 presents the previous studies to predict CKD.  Section 3 presents the main stages of a developing system to predict CKD based on Apache Spark.  Section 4 presents the experimental results. Finally, conclusions are presented in Section 5.

## 2. Related Works

Many authors have used different ML techniques for the diagnosis and prediction of chronic kidney disease as shown in Table 1.

For example, in [27], the authors proposed a hybrid model that combines LR and RF to predict CKD disease. They compared their proposed model with six ML algorithms, LR, RF, SVM, KNN, Naive Bayes (NB), and feedforward neural network (FNN). Their proposed model has registered the highest accuracy at 99.83%. In [29], NB, K-Star, SVM, and J48 classifiers were used to predict CKD. Performance comparison was made using WEKA software. J48 algorithm had better performance with 99% accuracy than the other algorithms.

Some authors used ML algorithms with feature selection methods to predict CKD. In [22], the recursive feature elimination (RFE) feature selection method has been used to select the essential features from the chronic kidney disease (CKD) dataset. Four classification algorithms have been applied (SVM, KNN, DT, and RF) to both full features and selected features. The results showed that RF outperformed all other algorithms. In [20], the authors used chi-square, CFS, and Lasso feature selection to select the essential features from the database. They applied ANN, C5.0, LR, LSVM, KNN, and RF to both full features and the selected features. The results showed that LSVM with full features has registered the highest accuracy at 98.86%. In [23], five feature selection methods, Random Forest feature selection (RF-FS), forward selection (FS), forward exhaustive selection (FES), backward selection (BS), and backward exhaustive (BE), have been used to select the most important features from the database. Four ML algorithms, RF, SVM, NB, and LR, have been used to predict CKD. The results showed that RF with Random Forest feature selection had achieved the best performance with 98.8% accuracy. In [26], the genetic search algorithm has been used to select the most important features from the CKD dataset. Decision Table, J48, Multilayer Perceptron (MLP), and NB have been applied to both full features and the selected features. Using genetic search algorithm enhanced the performance. The MLP classifier has achieved the best performance and outperformed the other classifiers. In [30], the number of important features has been selected using a correlation-based feature selection (CFS). AdaBoost, KNN, NB, and SVM have been used to detect CKD. The proposed CFS with AdaBoost achieved the best performance at 98.1% accuracy. In [25], the authors used two ensembles techniques which are Bagging and Random Subspace methods and three base-learners, KNN, NB, and DT, to predict CKD. The random subspace has achieved the best performance than Bagging on KNN classifier.

Previous studies just applied ML techniques to study and analyze data about CKD; they did not use big data platforms. Therefore, this motivates us to use big data platform (Spark) to study and analyze data about CKD including hybrid approaches (feature selection methods with ML classification algorithms and feature selection methods with ensemble algorithms).

## 3. Methodology

**Figure 1:**
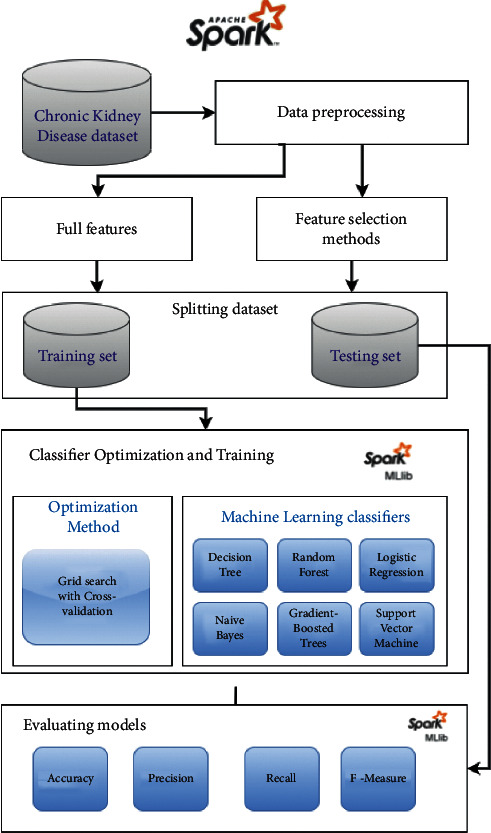
The steps of prediction CKD based on Apache Spark.

The proposed system of predicting chronic kidney disease consists of two main approaches, as shown in Figure 1. The first approach uses feature selection methods to select the essential features from the chronic kidney disease datasets. The second approach applies ML techniques: DT, LR, RF, SVM, NB, and ensemble learning on the selected features and full features to predict CKD. The proposed system is composed of 6 steps: in the first step (data collection), the CKD dataset from the UCI machine learning repository will be used. In the second step (data preprocessing step), null values will be handled. In the third step, the feature methods will be used to select the essential features. In the fourth step, a grid search with stratified cross-validation is used to optimize the parameters of ML and ensemble learning techniques. Each step is described in detail in the following subsections.

### 3.1. Data Collection

The chronic kidney disease (CKD) dataset used in this study was obtained from the UCI machine learning repository [31]. The CKD dataset includes 400 samples, 24 features, and 1 class label. The dataset contains 400 samples. The class label has two values: ckd (sample with CKD) and notckd (sample without CKD). The details of each feature are described in Table 2.

### 3.2. Data Preprocessing

The dataset included outliers and noise. Therefore, it needs to be cleaned up and unblemished in a preprocessing stage. The preprocessing stage incorporated the estimation of the missing values and noise elimination, like outliers, normalization, and unbalanced data checking, because certain measures may be lost when patients are being tested, resulting in missing values. There were 158 completed cases in the dataset, with the remainder occurrences having missing values. Ignoring the record is the simplest way of dealing with the missing values, although this strategy is ineffective in small datasets. Instead of removing records, we can apply algorithms to estimate the missing data as an alternative approach. The missing values of nominal features have been filled by mode. The missing values of numerical features have been filled by mean.

### 3.3. Feature Selection Methods

The main benefits of using feature selection algorithms are determining the important features in the dataset. The classifier approach with feature selection produces better results and reduces the model's execution time. Relief-F and chi-squared feature selection method were used to select the subset of important features from the database. This study has applied two feature selection strategies based on Apache Spark.

  RelieF [32] is a frequently used feature weighting technique that assigns weights to each feature in a dataset to determine the quality of the features [33]
  A chi-squared test is used a statistical hypothesis test to get ranks for each feature [18]

### 3.4. Splitting the Dataset

The CKD datasets are split into 80% training set and 20% testing set. We used stratified cross-validation to train and optimize the models using the training set and the result of cross-validation is registered. We evaluated the models using the testing set, and the results of the testing set are registered.

### 3.5. Models' Optimization and Training

#### 3.5.1. Optimization Methods

Grid search with stratified K-Fold cross-validation is used to optimize the models and tune the hyperparameters. The most common method for hyperparameter optimization is grid search. For each hyperparameter, the users must first define a set of values. The model then evaluates all possible values for each hyperparameter and chooses the one that provides the best performance.

K-Fold cross-validation: the dataset is divided into *k* folds of equal size. The training is done in k-1 groups, with the remaining time being used to test the classifiers. This procedure is repeated until each of the ten folds has been provided as a testing set. The performance of the classifiers is also measured for each *k*. Finally, depending on the average performance, the evaluation classifier is created.

#### 3.5.2. Machine Learning Models

The classification models used in the research are as follows:

  Decision tree (DT): it could be a supervised rule for learning in classification issues that contains a predefined target variable which is generally used. Decision tree works for each specific and continuous input and output variables. During this methodology, decision tree will be applied to each classification and regression issue that divides the population or sample into two or additional same sets known as subpopulation supporting the foremost necessary splitter within the input variable [34].
  Random forest (RF): it is a type of supervised ML technique. Basically, it accumulates a lot of trees and integrates them for more accurate prediction [23].
  Logistic regression (LR): it solved binary classification problems. A logistic or sigmoid function is used in LR to predict the probabilities of various labels for an unlabeled observation [35].
  Support vector machine (SVM): it is a type of supervised ML technique. It segregates dataset into classes using the hyperplane [22].
  Naïve Bayes (NB): the Bayes theorem is used to train a classifier in the Nave Bayes algorithm. In other words, it is a probabilistic classifier that has been trained using the Nave Bayes algorithm. It calculates a probability distribution over a set of classes for a given observation [29].
  Gradient-Boosted Trees (GBTs): it is also possible to train an ensemble of decision trees using the Gradient-Boosted Trees (GBTs) algorithm. However, each decision tree is trained sequentially. This makes use of the previously trained tree information to optimize each new tree. As a result, the model improves with every new tree. Since GBT trains one tree at a time, it can take longer time to train a model using GBT. In addition, if many trees are used in an ensemble, it is prone to overfitting. In a GBT ensemble, each tree can, however, be shallow, making it easier to train. Gradient boosting is a technique for iteratively training a series of decision trees. On each iteration, the method predicts the label of each training sample using the current ensemble and then compares the prediction to the true label [36].

### 3.6. Evaluating the Models

As shown in Equations 1-4, the models are evaluated using four standard metrics: accuracy, precision, recall, and F1-score, where TP stands for true positive, TN stands for true negative, FP stands for false positive, and FN stands for false negative.

(1) $$\begin{array}{c} \text{Accuracy}=\frac{\text{TP}+\text{TN}}{\text{TP}+\text{FP}+\text{TN}+\text{FN}}, \end{array}$$

(2) $$\begin{array}{c} \text{Precision}=\frac{\text{TP}}{\text{TP}+\text{FP}}, \end{array}$$

(3) $$\begin{array}{c} \text{Recall}=\frac{\text{TP}}{\text{TP}+\text{FN}}, \end{array}$$

(4) $$\begin{array}{c} F1=\frac{2\cdot \text{precision}\cdot \text{recall}}{\text{precision}+\text{recall}}. \end{array}$$

## 4. Experiments and Results

This section discusses the results of applying chi-square and Relief-F to the dataset to select the most important features. Also, it discusses the performance of cross-validation and the testing results of applying ML algorithms, SVM, LR, NB, RF, DT, and GBT Classifier, to the full features and the selected features. In addition, it demonstrates the best values of parameters for each ML algorithm that was optimized by grid search. Two feature selection methods were used; the CKD dataset was split into 80% training set and 20% testing set. The cross-validation results were registered for the training set, and the testing results were registered for the testing set. ML algorithms and features selection methods were implemented using PySpark.

### 4.1. Results of Chi-Square Feature Selection Method and ML Algorithms

**Figure 2:**
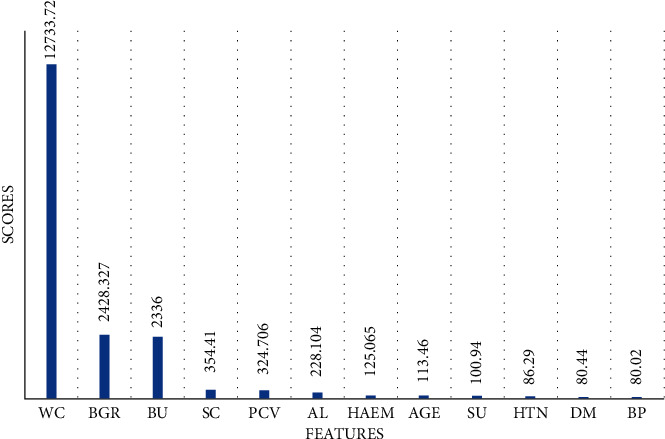
The important features selected by chi-square.

In this subsection, the essential features were selected by chi-square algorithm to pass into ML models for predicting CKD. The 12 most important features which have the highest scores and were thus used to predict CKD chi-square are wc, bgr, bu, sc, pcv, al, haem, age, su, htn, dm, and bp, as shown in Figure 2. It can be noticed that wc has the highest score at 12733.72, while bp has the lowest score at 80.02. The second highest score is registered by bgr at 2428.327. Sc and pcv have the same score at 354.410 and 324.706, respectively. Also, htn and dm have approximately the same score at 86.29 and 80.44, respectively.  Table 3 displays the scores of all features that chi-square has selected. The highest score is registered by wc at 12733.72, while the lowest is registered by sg at 0.0050.

The performance of cross-validation and the testing results of applying ML to the selected features by chi-square are described in Table 4. For cross-validation result, RF registered the highest performance (AC = 100%, PR = 100%, RE = 100%, FS = 100%), while NB has registered the lowest performance (AC = 81%, PR = 85%, RE = 82%, FS = 82%). LR and SVM have the same performance (AC = 97%, PR = 97%, RE = 97%, FS = 97%). For the testing results, SVM registered the highest performance (AC = 100%, PR = 100%, RE = 100%, FS = 100%), while NB registered the lowest performance (AC = 82%, PR = 88%, RE = 82%, FS = 82%). The second highest performance is registered by LR (AC = 97%, PR = 98%, RE = 97%, FS = 97%).

For optimization ML models, some of values of parameters are adapted and the best setting of ML's parameters is shown in Table 5.

### 4.2. Results of Relief-F Feature Selection Method and ML Algorithms

**Figure 3:**
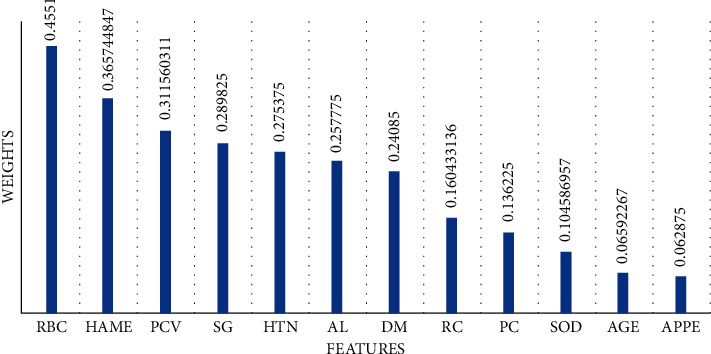
The weights of the most essential selected by Relief-F.

In this subsection, the essential features were selected by Relief-F algorithm to pass into Ml models for predicting CKD. The 12 most important features which have the highest weights selected by Relief-F and were used to predict CKD are shown in Figure 3. It can be noticed that rbc has the highest weight at 0.4551, while appe has the lowest weight at 0.062875. The second highest weight is registered by haem at 0.365745. Al and dm have approximately the same weights at 0.257775 and 0.24085, respectively.

Table 6 displays weights of all features that are selected by Relief-F. The highest weight is registered by rbc at 0.4551, while the lowest weight is registered by bp at -0.01584. The performance of cross-validation and the testing results of applying ML to the features selected by Relief-F are described in Table 7. For cross-validation results, DT, RF, and GBT Classifier registered the highest performance (AC = 100%, PR = 100%, RE = 100%, FS = 100%), while NB registered the lowest performance (AC = 88%, PR = 89%, RE = 89%, FS = 89%). LR and SVM have the same performance (AC = 99%, PR = 99%, RE = 99%, FS = 99%).

For the testing results, DT and GBT Classifier registered the highest performance (AC = 100%, PR = 100%, RE = 100%, FS = 100%), while NB registered the lowest performance (AC = 95%, PR = 95%, RE = 95%, FS = 95%). LR and SVM have the same performance (AC = 98%, PR = 99%, RE = 99%, FS = 99%).

For optimization ML models, some of values of parameters are adapted and the best setting of ML's parameters is shown in Table 8.

### 4.3. The Performance of ML with Full Features

Table 9 presents the result of cross-validation and the testing of applying ML to full features. Overall, RF achieved the best performance for cross-validation and the testing results. For cross-validation results, RF registered the highest performance (AC = 100%, PR = 100%, RE = 100%, FS = 100%), while NB has the lowest performance (AC = 84%, PR = 88%, RE = 84%, FS = 84%). LR, SVM, and GBT Classifier have the same performance (AC = 99%, PR = 99%, RE = 99%, FS = 99%). For the testing results, RF and SVM registered the highest performance (AC = 100%, PR = 100%, RE = 100%, FS = 100%), while NB has the lowest performance (AC = 87%, PR = 91%, RE = 88%, FS = 88%). For optimization ML models, some of values of parameters are adapted and the best setting of ML's parameters is shown in Table 10.

### 4.4. Discussion

Table 11 presents models that have achieved the highest cross-validation results. The performance of cross-validation of applying ML to the features selected by Relief-F has achieved the best value by three models: DT, RF, and GBT Classifiers. In comparisons, the cross-validation performance of applying ML to full features and features selected by chi-square has achieved the best value by 1 model: RF.

Table 12 presents the best models for the testing results. The performance of testing applying ML to the features selected by Relief-F has achieved the best value by two models: DT and GBT Classifiers. The testing performance of applying ML to full features has achieved the best value by two models: RF and SVM Classifiers. However, the testing performance of applying ML to the features selected by chi-square has achieved the best value by 1 model: SVM Classifier.

The results showed that SVM, DT, and GBT Classifier with the selected features have achieved the best performance. Overall, the performance with Relief-F feature selection is better than chi-square feature selection and full features.

Table 13 presents the comparison of performance between the previous studies and our work on the same dataset. In our work, the Relief-F feature selection methods have achieved the best performance for the testing results and cross-validation results using DT and GBT Classifier compared to the other existing works [23, 24, 26, 27, 30]. Also, our work is different from the other existing works [22, 25] because it registered the results for both the training set and the testing set, and it has achieved the best performance.

## 5. Conclusion

In this paper, the hybrid ML techniques integrating feature selection methods and classification ML algorithms based on big data platforms (Apache Spark) were used to predict CKD. Relief-F and chi-squared feature selection techniques were used to select the important features from the dataset. ML algorithms, DT, LR, NB, RF, SVM, and GBT Classifier as ensemble learning algorithm, were applied to benchmark chronic kidney disease dataset. Also, they were applied to the full features and to the selected features. Grid search with cross-validation was used to optimize the parameters of ML. In addition. Four methods of evaluation, accuracy, precision, recall, and F1-measure, were applied to validate the results and the results of cross-validation and the testing data were registered. The results showed that SVM, DT, and GBT Classifier with the selected features have achieved the best performance. Overall, the performance of Relief-F feature selection is better than that achieved by chi-square feature selection and the full features.

## Figures and Tables

**Table 1:** Related works for prediction of CKD.

REF	Year	Models	Feature selection methods	Dataset

[22]	2021	SVM, KNN, DT, and RF	Recursive feature elimination (RFE)	CKD dataset
[20]	2020	ANN, C5.0, and LR	CFS, Lasso, and	CKD dataset
		LSVM, KNN, and RF	Wrapper method	
[23]	2020	RF, SVM, NB, and LR	RF-FS, FS, FES, BS, and BES	CKD dataset
[24]	2020	An ensemble of decision tree models	Cost-sensitive ensemble	CKD dataset
			Feature ranking	
[25]	2020	Bagging and random subspace	No	CKD dataset
		Methods based on KNN		
		NB and DT		
[26]	2020	Decision Table, J48	Genetic search algorithm	CKD dataset
		MLP and NB		
[27]	2019	LR, RF, SVM, KNN	No	CKD dataset
		NB and FNN		
		A hybrid model LR and RF		
[28]	2019	Artificial neural network (ANN) and SVM	Correlation coefficients	CKD dataset
[29]	2018	NB and K-Star	No	CKD dataset
		SVM		
		J48		
[30]	2018	AdaBoost and KNN	CFS	CKD dataset
		NB and SVM		

**Table 2:** The CKD dataset description.

Features	Explain

age	Age
bp	Blood pressure
sg	Specific gravity
al	Albumin
su	Sugar
rbc	Red blood cells
pc	Pus cell
pcc	Pus cell clumps
ba	Bacteria
bgr	Blood glucose random
bu	Blood urea
sc	Serum creatinine
sod	Sodium
pot	Potassium
hemo	Hemoglobin
pcv	Packed cell volume
wc	White blood cell count
rc	Red blood cell count
htn	Hypertension
dm	Diabetes mellitus
cad	Coronary artery disease
appet	Appetite
pe	Pedal edema
ane	Anemia
class	Class

**Table 3:** The scores of all features that are selected by chi-square.

Features	Scores

wc	12 733.72
bgr	2428.327
bu	2336.00
sc	354.410
pcv	324.706
al	228.104
haem	125.065
age	113.460
su	100.94
htn	86.29
dm	80.44
bp	80.02
pe	45.10
ane	35.611
sod	28.793
pcc	24.075
rc	20.84
cad	19.93
pc	14.16
ba	12.58
appe	12.58
rbc	9.41
pot	4.07
sg	0.0050

**Table 4:** The performance of ML with the features selected by chi-square.

Models	Cross-validation performance				Test performance			
	AC	PR	RE	FS	ACC	PR	RE	FS

DT	97	98	98	98	92	93	93	93
RF	100	100	100	100	95	95	95	95
LR	97	97	97	97	97	98	97	97
SVM	97	97	97	97	100	100	100	100
NB	81	85	82	82	82	88	82	82
GBT Classifier	98	98	98	98	95	95	95	95

**Table 5:** The best values of ML's parameters are applied to the features selected by chi-square.

Model	Parameters	Values

DT	Impurity	Gini
	maxDepth	3
	maxBins	10

RF	Impurity	Gini
	maxDepth	6
	maxBins	32

LR	regParam	0.8
	maxIter	20

SVM	regParam	0.01
	maxIter	100
NB	Smoothing	0.2

GBT Classifier	maxDepth	2
	maxBins	60

**Table 6:** The performance of ML with the features selected by Relief-F.

Models	Cross-validation performance				Test performance			
	AC	PR	RE	FS	AC	PR	RE	FS

DT	100	100	100	100	100	100	100	100
RF	100	100	100	100	98	99	99	99
LR	99	99	99	99	98	99	99	99
SVM	99	99	99	99	98	99	99	99
NB	88	89	89	89	95	95	95	95
GBT Classifier	100	100	100	100	100	100	100	100

**Table 7:** The best values of ML's parameters which are applied to the features selected by Relief-F.

Model	Parameters	Values

DT	Impurity	Gini
	maxDepth	4
	maxBins	32

RF	Impurity	Gini
	maxDepth	5
	maxBins	32

LR	regParam	0.1
	maxIter	20

SVM	regParam	0.01
	maxIter	100
NB	Smoothing	0.1

GBT Classifier	maxDepth	4
	maxBins	20

**Table 8:** The weights of all features that are selected by Relief-F.

Features	Weights

rbc	0.455 1
haem	0.365 745
pcv	0.311 56
sg	0.289 825
htn	0.275 375
al	0.257 775
dm	0.240 85
rc	0.160 433
pc	0.136 225
sod	0.104 587
Age	0.065 923
appe	0.062 875
pe	0.056 825
su	0.031 65
bgr	0.029 549
ane	0.027
bu	0.022 733
sc	0.015 806
pcc	0.015 675
wc	0.006 426
ba	−0.000 12
pot	−0.004 11
cad	−0.011 97
bp	−0.015 84

**Table 9:** The performance of ML with full features.

Models	Cross-validation performance				Test performance			
	AC	PR	RE	FS	AC	PR	RE	FS
DT	98.43	98	98	98	95	95	95	95
RF	100	100	100	100	100	100	100	100
LR	99	99	99	99	98	99	99	99
SVM	99	99	99	99	100	100	100	100
NB	84	88	84	84	87	91	88	88
GBT Classifier	99	99	99	99	95	95	95	95

**Table 10:** The best values of ML's parameters which are applied to full features.

Model	Parameters	Values

DT	Impurity	Gini
	maxDepth	4
	maxBins	10

RF	Impurity	Gini
	maxDepth	7
	maxBins	32

LR	regParam	0.3
	maxIter	10

SVM	regParam	0.01
	maxIter	1000
NB	Smoothing	0.2

GBT Classifier	maxDepth	2
	maxBins	60

**Table 11:** Best models for cross-validation results.

Best models	Features	Measure methods			
		AC	PR	RE	FS

RF	Full features	100	100	100	100
RF	Features selected by chi-square	100	100	100	100
DT	Features selected by Relief-F	100	100	100	100
RF	Features selected by Relief-F	100	100	100	100
GBT Classifier	Features selected by Relief-F	100	100	100	100

**Table 12:** Best models for the testing results.

Best models	Features	Measure methods			
		AC	PR	RE	FS

SVM	Full features	100	100	100	100
RF	Full features	100	100	100	100
SVM	Features selected by chi-square	100	100	100	100
DT	Features selected by Relief-F	100	100	100	100
GBT Classifier	Features selected by Relief-F	100	100	100	100

**Table 13:** The comparison of performance between the previous studies and our work on the same dataset.

REF	Feature selection methods	The best model	Dataset	Result

[22]	RFE	RF	CKD dataset	AC = 100%
				PR = 100%
				RE = 100%
				FS = 100%
[27]	No	A hybrid model LR and RF	CKD dataset	AC = 99.94%
				*E* = 99.84%
				*S* = 99.80%
[30]	CFS	AdaBoost based on KNN	CKD dataset	AC = 98.1%
				PR = 98%
				RE = 98%
				FS = 98%
[23]	Rffs, FS, FES, BS, BES	RF	CKD dataset	AC = 98.825%
				RE = 98.04%
[24]	Cost-sensitive ensemble feature ranking	An ensemble of decision tree models	CKD dataset	AC = 97.27%
				PRC = 99.44%
				RE = 96.25%
				FS = 97.68%
[25]	No	Random subspace-based KNN	CKD dataset	AC = 100%
				RE = 100%
[26]	Genetic search algorithm	Multilayer perceptron	CKD dataset	AC = 99.75%
Our work	Relief-F	DT	CKD dataset	Cross-validation result AC = 100%, PRC = 100%, RRE = 100% FS = 100% result of testing AC = 100%, PRC = 100%, RRE = 100%, FS = 100%
		GBT Classifier	CKD dataset	Cross-validation result AC = 100%, PRC = 100%, RRE = 100%, FS = 100%; result of testing AC = 100%, PRC = 100%, RRE = 100%, FS = 100%

## Data Availability

Chronic kidney disease dataset is downloaded from https://archive.ics.uci.edu/ml/datasets/chronic_kidney_disease.

## References

[1] Manogaran G., Lopez D. (2018). Health data analytics using scalable logistic regression with stochastic gradient descent. *International Journal of Advanced Intelligence Paradigms*.

[2] Hu H., Wen Y., Chua T. S., Li X. (2014). Toward scalable systems for big data analytics: a technology tutorial. *IEEE access*.

[3] (2021). Apache Hadoop. https://hadoop.apache.org/.

[4] Kafka A. (2021). Apache Kafka. https://kafka.apache.org/.

[5] Storm A. (2021). Apache Storm. https://storm.apache.org/.

[6] Beam A. L., Kohane I. S. (2018). Big data and machine learning in health care. *JAMA*.

[7] Nair L. R., Shetty S. D., Shetty S. D. (2018). Applying spark based machine learning model on streaming big data for health status prediction. *Computers & Electrical Engineering*.

[8] Ali A. A. (2019). Stroke prediction using distributed machine learning based on Apache spark. *Stroke*.

[9] World Health Organization (2005). *Preventing Chronic Diseases: A Vital Investment*.

[10] Bikbov B., Perico N., Remuzzi G. (2018). Disparities in chronic kidney disease prevalence among males and females in 195 countries: analysis of the global burden of disease 2016 study. *Nephron*.

[11] Disease K. (2009). Improving global outcomes (kdigo) transplant work group. kdigo clinical practice guideline for the care of kidney transplant recipients. *American Journal of Transplantation*.

[12] Cdc (2021). Chronic Kidney Disease in the united states. https://www.cdc.gov/kidneydisease/publications-resources/ckd-national-facts.html.

[13] Deng L., Li X. (2013). Machine learning paradigms for speech recognition: an overview. *IEEE Transactions on Audio Speech and Language Processing*.

[14] Huang M. Q., Ninić J., Zhang Q. B. (2021). Bim, machine learning and computer vision techniques in underground construction: current status and future perspectives. *Tunnelling and Underground Space Technology*.

[15] Oza P., Sharma P., Patel S. (2021). Machine learning applications for computer-aided medical diagnostics. *Proceedings of the Second International Conference on Computing, Communications, and Cyber-Security*.

[16] Bikmukhametov T., Jäschke J. (2020). Combining machine learning and process engineering physics towards enhanced accuracy and explainability of data-driven models. *Computers & Chemical Engineering*.

[17] Palma-Mendoza R. J., Rodriguez D., Marcos L. D. (2018). Distributed relieff-based feature selection in spark. *Knowledge and Information Systems*.

[18] Nassar M., Safa H., Mutawa A. A., Helal A., Gaba I. Chi squared feature selection over Apache spark.

[19] Charleonnan A., Fufaung T., Niyomwong T., Chokchueypattanakit W., Suwannawach S., Ninchawee N. Predictive analytics for chronic kidney disease using machine learning techniques.

[20] Chittora P., Chaurasia S., Chakrabarti P. (2021). Prediction of chronic kidney disease-a machine learning perspective. *IEEE Access*.

[21] Spark A. (2021). *Apache Spark*.

[22] Senan E. M., Adhaileh M. H. A., Alsaade F. W. (2021). Diagnosis of chronic kidney disease using effective classification algorithms and recursive feature elimination techniques. *Journal of Healthcare Engineering*.

[23] Abdullah A. A., Hafidz S. A., Khairunizam W. (2020). Performance comparison of machine learning algorithms for classification of chronic kidney disease (ckd). *Journal of Physics: Conference Series*.

[24] Ali S. I., Ali B., Hussain J. (2020). Cost-sensitive ensemble feature ranking and automatic threshold selection for chronic kidney disease diagnosis. *Applied Sciences*.

[25] Jongbo O. A., Adetunmbi A. O., Ogunrinde R. B., Ajisafe B. B. (2020). Development of an ensemble approach to chronic kidney disease diagnosis. *Scientific African*.

[26] Jena L., Patra B., Nayak S., Mishra S., Tripathy S. (2021). Risk prediction of kidney disease using machine learning strategies. *Intelligent and Cloud Computing*.

[27] Qin J., Chen L., Liu Y., Liu C., Feng C., Chen B. (2019). A machine learning methodology for diagnosing chronic kidney disease. *IEEE Access*.

[28] Almansour N. A., Syed H. F., Khayat N. R. (2019). Neural network and support vector machine for the prediction of chronic kidney disease: a comparative study. *Computers in Biology and Medicine*.

[29] Avci E., Karakus S., Ozmen O., Avci D. Performance comparison of some classifiers on chronic kidney disease data.

[30] Wibawa M. S., Maysanjaya I. M. D., Putra I. M. A. W. Boosted classifier and features selection for enhancing chronic kidney disease diagnose.

[31] Repository U. M. L. (2021). Chronic kidney disease data set. https://archive.ics.uci.edu/ml/datasets/chronic_kidney_disease.

[32] Kononenko I. Estimating attributes: analysis and extensions of relief.

[33] Canedo V. B., Remeseiro B. (2017). Sanchez-maro no, n., and alonso-betanzos. *Transactions on Computational Collective Intelligence XX*.

[34] Chaurasia V., Pal S., Tiwari B. (2018). Chronic kidney disease: a predictive model using decision tree. *International Journal of Engineering Research and Technology*.

[35] Haq A. U., Li J. P., Memon M. H., Nazir S., Sun R. (2018). A hybrid intelligent system framework for the prediction of heart disease using machine learning algorithms. *Mobile Information Systems*.

[36] Shi H., Wang H., Huang Y., Zhao L., Qin C., Liu C. (2019). A hierarchical method based on weighted extreme gradient boosting in ecg heartbeat classification. *Computer Methods and Programs in Biomedicine*.

